# Glutamine enhances endothelial cell survival and vasodilation by increasing glutathione to reduce oxidative stress

**DOI:** 10.14814/phy2.70737

**Published:** 2026-01-21

**Authors:** Marzyeh Kheradmand, Gurneet Sangha, Claire M. Sissons, Michael Sun, Xinyao Zhou, Lauren V. Smith, Meagan Bauer, Chengpeng Chen, Alisa Morss Clyne

**Affiliations:** ^1^ Fischell Department of Bioengineering University of Maryland College Park Maryland USA; ^2^ Department of Chemistry and Biochemistry University of Maryland Baltimore County Baltimore Maryland USA

**Keywords:** cardiovascular disease, glutamine, hyperglycemia, oxidative stress

## Abstract

Cardiovascular disease is exacerbated by diabetes through hyperglycemia‐induced endothelial dysfunction, which arises from oxidative stress. Glutamine is postulated to decrease oxidative stress; however, its effect on endothelial dysfunction in hyperglycemia is unknown. Therefore, we investigated how glutamine affects endothelial function in normal and high glucose. Human coronary artery endothelial cells were treated with 0, 0.5, or 2 mM glutamine in 5.5 or 15 mM glucose for 24 h. We then assessed cell proliferation, oxidative stress, cell survival, and endothelial nitric oxide synthase (eNOS) activity. Our data showed that independent of glucose concentration, glutamine increased proliferation by up to 3.5‐fold. Furthermore, glutamine metabolism through glutaminase‐1 reduced oxidative stress and cell death by up to 70% and 94%, respectively, by doubling glutathione and NADPH. Glutamine also increased ex vivo vasodilation in isolated murine carotid arteries without altering eNOS activity or nitric oxide in vitro, suggesting that the enhanced vasodilation results from reduced oxidative stress. These findings indicate that glutamine mitigates endothelial cell oxidative stress by enhancing reducing capacity, which may protect against diabetic cardiovascular disease.

## 1 INTRODUCTION

Cardiovascular disease (CVD) is the leading cause of morbidity and mortality in individuals with diabetes. Hyperglycemia, which is a characteristic of both type 1 and type 2 diabetes, plays a critical role in endothelial dysfunction, a key initiating step in CVD. Endothelial cells exposed to hyperglycemia have reduced nitric oxide bioavailability (Montezano & Touyz, 2012), dysregulated angiogenesis (Pipino et al., 2022), increased inflammatory adhesion molecule expression (Mansour et al., 2015), and elevated permeability (Taïlé et al., 2021). Hyperglycemia changes endothelial cell function through metabolic alterations, including increased glucose flux through the polyol and hexosamine biosynthetic (HBP) pathways as well as through the mitochondrial tricarboxylic acid (TCA) cycle (Du et al., 2000). When more glucose‐derived pyruvate is oxidized in the TCA cycle, more mitochondrial superoxide is produced which, in turn, can activate other superoxide production pathways (Du et al., 2001; Kassab & Piwowar, 2012). Many of the aforementioned aspects of endothelial cell dysfunction observed in hyperglycemic conditions and diabetes are linked to elevated oxidative stress.

Endothelial cells also metabolize glutamine, the most abundant amino acid in the human body, and glutamine metabolism has the potential to ameliorate endothelial dysfunction induced by hyperglycemia. Glutamine is first metabolized by glutaminase‐1 (GLS1), which converts glutamine to glutamate and ammonia. Glutamate can then be converted to α‐ketoglutarate and enter the TCA cycle, or glutamate can be converted to the antioxidant glutathione (GSH) (Huang et al., 2017; Kim et al., 2017; Peyton et al., 2018; Zhao et al., 2025). Glutamine is required for endothelial cell proliferation, as endothelial cells cultured without glutamine or with GLS1 knocked down have significantly reduced proliferation and angiogenesis (Durante, 2019; Huang et al., 2017; Kim et al., 2017; Peyton et al., 2018).

However, reported effects of glutamine on nitric oxide and vasodilation are contradictory. In vitro, glutamine impairs recycling of L‐arginine, a key substrate for nitric oxide production by endothelial nitric oxide synthase (eNOS). Glutamine also increases HBP flux, which can lead to eNOS O‐GlcNAcylation and subsequent impaired activation through phosphorylation at serine 1177 (Arnal et al., 1995; Basehore et al., 2021; Kawaguchi et al., 2005; Peyton et al., 2018; Wu et al., 2001). In vivo, glutamine increases vasodilation in most animal and human studies. For example, glutamine supplementation partially restored vasodilation in mice with induced endothelial dysfunction, and 6 months of glutamine supplementation enhanced flow‐mediated vasodilation in older adults (Addabbo et al., 2013; Ellis et al., 2016).

Several studies have shown the potential of glutamine as a therapeutic for diabetic CVD. Glutamine levels are reduced in the blood of people with diabetes (Mansour et al., 2015; Zhao et al., 2025). Reduced GLS1 expression was found in skin wounds and cardiac endothelial cells from diabetic patients (Zhao et al., 2025). In vitro, glutamine reduced mitochondrial dysfunction in human umbilical vein endothelial cells cultured in high glucose, while supplementation with α‐ketoglutarate and nonessential amino acids restored proliferation and tube formation in endothelial cells cultured in both high glucose and high fat (Safi et al., 2015; Zhao et al., 2025). In vivo, oral glutamine supplementation significantly improved CVD risk factors in patients with type 2 diabetes, including those linked to endothelial dysfunction such as systolic blood pressure (Mansour et al., 2015).

Glutamine supplementation has therapeutic potential to ameliorate endothelial dysfunction caused by high glucose. However, the mechanisms by which glutamine impacts hyperglycemia‐induced endothelial dysfunction are not well understood. We hypothesized that glutamine promotes endothelial cell survival and vasodilation by increasing the biosynthesis and recycling of the antioxidant glutathione to reduce hyperglycemia‐induced oxidative stress. To investigate this hypothesis, we first measured proliferation, oxidative stress, and survival when primary human coronary artery endothelial cells were cultured in varied glutamine levels in normal and high glucose. We further measured vasodilation ex vivo in murine carotid arteries and nitric oxide production in endothelial cells in vitro in the presence or absence of glutamine. We then measured the antioxidant glutathione and the reducing agent NADPH in cells cultured in varied glutamine levels in normal and high glucose. Finally, we chemically inhibited GLS1 to determine if glutamine conversion to glutamate enhanced endothelial cell survival by regulating oxidative stress through glutathione. These studies deepen our understanding of how glutamine metabolism impacts oxidative stress and nitric oxide in endothelial cells in high glucose, revealing new strategies to reduce CVD in diabetic patients.

## 2 MATERIALS AND METHODS

### 2.1 Cell culture

Primary human coronary artery endothelial cells (HCAEC, passage 5–9) were purchased from Lifeline Cell Technology. HCAECs were cultured in Endothelial Growth Medium‐2 (EGM‐2; Lonza) supplemented with 1% penicillin‐streptomycin (PS; ThermoFisher, 15140163), 10% fetal bovine serum (FBS; Cytiva, SH30088), and 2 mM glutamine (Fisher Scientific, 25‐030‐081) until 90% confluent. The growth medium was then removed, cells were rinsed with phosphate buffered saline (PBS, ThermoFisher, 70011069), and Dulbecco's Modified Eagle Medium (DMEM without phenol red, glucose, or glutamine, Gibco, A1443001) supplemented with EGM‐2 Endothelial SingleQuots (Lonza, CC‐4176), 5.5 or 15 mM D‐glucose (Sigma‐Aldrich, G8270), and 0, 0.5, or 2 mM glutamine was added for 24 h. These glucose and glutamine concentrations were selected to model physiologically relevant and common experimental conditions. Specifically, 5.5 mM glucose represents normal plasma glucose, whereas 15 mM glucose represents hyperglycemia. 0 mM glutamine represents glutamine depletion, 0.5 mM glutamine represents physiological plasma glutamine, and 2 mM glutamine represents supplemented endothelial cell culture media (Lee et al., 2011; Low et al., 1993; Wals & Katz, 1993). Media with no glutamine and L‐glucose (Sigma‐Aldrich, G5500) at 2 or 11.5 mM was the osmotic control (OC) for glutamine in 5.5 mM glucose media and glucose and glutamine in 15 mM glucose media, respectively. Bis‐2‐(5‐phenylacetamido‐1,2,4‐thiadiazol‐2‐yl)ethyl sulfide (BPTES, Sigma‐Aldrich, SML0601, 10 μM) was used to inhibit glutamine metabolism to glutamate. BPTES binds to an allosteric site on GLS1 to keep it in an inactive confirmation, thereby inhibiting glutamine hydrolysis (Shukla et al., 2012).

### 2.2 Cell viability

Endothelial cell viability was assessed using ethidium homodimer (EthD‐1, ThermoFisher, E1169), which enters permeabilized dead cells and fluoresces red after it binds to DNA. We used Hoechst to count the total number of cells (live and dead), since the live cell label Calcein AM fluoresced even in dead cells due to ROS (Uggeri et al., 2000). To assess viability in response to stress, cells were cultured in varied glucose and glutamine media for 24 h and then stressed with 50 μM tert‐butyl hydroperoxide (tBHP; Sigma‐Aldrich, 458139), an inducer of oxidative stress (Wedel et al., 2020) for another 24 h. After a PBS wash, 2 μM EthD‐1 and Hoechst (ThermoFisher, 62249, 1:2000) were added in DMEM supplemented with 10% FBS and 1% PS for 30 min at room temperature. 50% methanol was added for 10 min to some wells as a positive control for cell death. After washing with PBS, cells were imaged using a Nikon Eclipse Ti2 spinning disk confocal microscope at 10× magnification. Nuclei (blue) and dead (red) cells were quantified using NIS software, and the percentage of dead cells was calculated as: $\frac{\text{number of dead cells}}{\text{total number of nuclei}}\times 100$.

### 2.3 Cell proliferation

Cell proliferation was assessed by labeling for Ki67, a protein found in the nucleus of actively dividing cells, as well as cell counts. For Ki67 labeling, HCAECs were seeded at 75,000 cells per well on glass coverslips in 12‐well plates and allowed to adhere overnight. Cells were serum‐starved in EBM‐2 with 1% PS for 24 h, followed by treatment with varied glucose and glutamine media. Human fibroblast growth factor‐2 (hFGF‐2; Peprotech, 100‐18B) at 1 ng/mL served as a positive control. After 24 h, cells were fixed in 4% paraformaldehyde (Millipore Sigma, P6148) for 15 min and then permeabilized and blocked in 0.2% Triton X‐100 (Alfa Aesar, A16046) with 0.5% BSA (Sigma‐Aldrich, A7906) in PBS for 40 min. A Ki67 primary antibody (Abcam, ab15580) was applied overnight at 4°C (1:200 in 0.2% Triton X‐100 with 0.5% BSA in PBS). The next day, cells were incubated for 1 h with donkey anti‐rabbit IgG Alexa Fluor 488‐conjugated secondary antibody (ThermoFisher, A‐21206, 1:1000) and DAPI (ThermoFisher, 62248, 1:2000). Samples were rinsed with PBS and coverslips were mounted and imaged on a Revolve microscope (ECHO) at 20× magnification. Ki67 co‐localization with nuclei was quantified using a custom Python script. The percentage of Ki67‐positive cells was calculated as: $\frac{\#\text{of}\;\mathrm{ki}67\;\text{positive nuclei}}{\text{total}\#\text{of nuclei}}\times 100$.

For cell counts, HCAECs were seeded at 50,000 cells per well in a 24‐well plate in varied glucose and glutamine media. After 2 days, cells were detached using trypsin (ThermoFisher, 25300120) and counted using a Countess II FL (ThermoFisher). hFGF‐2 (1 ng/mL) was included as a positive control.

### 2.4 Reactive oxygen species (ROS)

5‐(and‐6)‐chloromethyl‐2′,7′‐dichlorodihydrofluorescein diacetate, acetyl ester (CM‐H_2_DCFDA, Invitrogen, C6827), which becomes fluorescent after oxidation by intracellular ROS, was used as a general ROS indicator. HCAEC cultured in varied levels of glucose and glutamine for 24 h were treated with 2 μM CM‐H_2_DCFDA and Hoescht (1:2000, to visualize nuclei) in DMEM for 1 h at 37°C. Cells treated with 0.5 mM tBHP in EGM‐2 media for 1 h were the positive control. Cells were then washed with PBS and imaged on a Nikon Eclipse Ti2 spinning disk confocal microscope with a 10× objective. After rolling ball background subtraction, mean fluorescence intensity was quantified in ImageJ and normalized to the number of nuclei.

### 2.5 Glutathione

Intracellular glutathione was measured using a GSH/GSSG Glo assay (Promega, V6612) and liquid chromatography‐mass spectrometry (LC‐MS). For the Glo assay, HCAEC were seeded in a white bottom 96‐well polystyrene plate (Sigma‐Aldrich, CLS3917) at 50,000 cells/well. The next day, cells were treated with varied glucose and glutamine media for 24 h. On the day of the assay, cells were rinsed with Hanks' Balanced Salt solution (HBSS, Millipore Sigma, 55037C) and then lysed for 30 min at room temperature in the presence of luciferin NT substrate and glutathione S‐transferase enzyme. Luciferin detection reagent was then added for 15 min, after which luminescence was measured using a Spark multimode plate reader. Glutathione concentration was determined using a standard curve.

For LC‐MS, metabolites were extracted from confluent HCAECs treated with varied glucose and glutamine media for 24 h using 80% HPLC grade methanol (Sigma‐Aldrich, G6025‐11VL) and 20% HPLC grade water (Midland Scientific, Wooo6‐4L) at −80°C for 15 min. Cells were then scraped, transferred to 1.5 mL centrifuge tubes (Cell Treat, 229443), and centrifuged at 17,000 × *g* for 10 min. The supernatant was transferred to a new 1.5 mL tube, and the cell pellet was stored at −80°C for protein concentration measurement. The supernatant was dried using a CentriVap Vacuum Concentrator (Labconco), resuspended in 200 μL 5% HPLC grade methanol and 95% HPLC grade water, and transferred to mass spectrometry vials (Waters, 600000670). Quality control samples were prepared by adding 50 μL of each sample to a separate vial. Reduced L‐Glutathione (GSH, Sigma‐Aldrich, G4251) was used to establish a standard curve. 3 μL of each sample was analyzed in duplicate using a Shimadzu LCMS‐8045 CL Triple Quad LC–MS/MS with an attached Nexera HPLC/UHPLC Pump (LC‐20AD XR) and a Luna Omega Polar C18 reverse‐phase column (2.1 × 50 mm, 1.6 μm; Phenomenex). Mobile phases consisted of HPLC grade water with 1% formic acid (mobile phase A) and HPLC grade acetonitrile with 1% formic acid (mobile phase B). The flow rate was maintained at 0.45 mL/min. A gradient elution was applied as follows: 5% B for 0.6 min, linearly increased to 20% B at 1.80 min, ramped to 50% B at 1.81 min, and then linearly increased to 100% B from 3.00 to 5.00 min. The column was re‐equilibrated with 5% B from 6.00 to 8.00 min. Mass spectrometry was performed in positive electrospray ionization mode (ESI+) with a capillary voltage of 4 kV. Nebulizing gas and drying gas flows were set to 3 L/min and 10 L/min, respectively. Desolvation and interface temperatures were set to 250°C and 300°C. GSH was quantified using multiple reaction monitoring (MRM) with a mass transition of m/z 307.9 → 179. Data were processed in Skyline Software for peak identification and ion count integration.

### 2.6 NADPH/NADP+ ratio

The NADP/NADPH‐Glo assay (Promega, G9082) was used to measure the NADPH/NADP+ ratio, which is one indication of intracellular reducing capacity. Briefly, confluent HCAECs in a white bottom 96‐well polystyrene plate were treated with varied glucose and glutamine media. After 24 h, the cells were lysed in 1% dodecyl trimethyl azanium bromide (Sigma, D‐8638‐25). 50 μL cell lysate was then transferred to an empty well and treated with 25 μL 0.4 N HCl to measure NADP^+^, while the base only wells were used to measure NADPH. Each group was mixed with an equal volume of NADP/NADPH‐Glo detection reagents. After 30 min, luminescence was recorded using a Spark multimode plate reader, and the NADPH/NADP^+^ ratio was calculated.

### 2.7 Pressure myography

The animal protocol was approved by the University of Maryland Institutional Animal Care and Use Committee (IRBNet ID: 1932985‐6). Twelve‐week‐old male (*n* = 5) and female (n = 5) C57BL/6J mice were obtained from Jackson Laboratory (Bar Harbor, ME) and allowed to acclimate for 3 days. Standard chow diet was provided ad libitum. Mice were euthanized by exsanguination. The carotid arteries were excised and placed in cold HEPES physiological saline solution (HEPES‐PSS) supplemented with either 0 or 2 mM glutamine for 24 h at 4°C.

For pressure myography, the carotid artery was cannulated with stainless steel micropipettes in a pressure myograph chamber (114P; DMT) containing cold HEPES‐PSS. The myograph chamber was slowly warmed to 37°C and aerated with carbogen (5% CO_2_, 95% O_2_). Arterial pressure was increased from 0 to 40 mmHg at increments of 10 mmHg every 5 min. The carotid artery was then equilibrated at 50 mmHg for an additional 15 min. Vessel viability was confirmed by superfusing arteries with high potassium PSS (KPSS) for 5 min. Carotid arteries were considered viable if at least 10% constriction was achieved. Carotid arteries were then submaximally constricted 10%–15% using 10^−5^ M phenylephrine (61‐76‐7; Acros Organics) prior to vasodilation experiments. Vasodilation was measured by treating carotid arteries with 10^−9^ M to 10^−5^ M acetylcholine (A6625; Sigma‐Aldrich). Endothelial‐independent vasodilation was measured by preincubating carotid arteries with eNOS inhibitor NG‐Nitroarginine methyl ester hydrochloride (L‐NAME, 10^−5^ M; HY‐18729A; Med Chem Express) for 1 h. Vasodilation to acetylcholine was then measured as previously described.

### 2.8 Nitrate/nitrite

Nitrates and nitrites in endothelial cell media were measured as a nitric oxide byproduct using the Nitrate/Nitrite Colorimetric Assay Kit (Cayman Chemical, 780,001) as per manufacturer instructions. Confluent HCAECs were treated with varied glucose and glutamine media. After 24 h, 40 μL media from each condition was added to a 96‐well plate in duplicate and mixed with nitrate reductase to convert nitrates into nitrites. Plates were incubated for 2 h at room temperature, followed by addition of Griess reagents for 10 min. Absorbance was measured at 550 nm using a Spark multimode plate reader (TECAN), and a standard curve was used to obtain nitrite concentration.

### 2.9 Statistical analysis

GraphPad Prism 10 was used to analyze all data. Due to small sample sizes (*n* < 30), we assumed non‐normal distributions for all datasets and used nonparametric statistical tests. When two conditions were compared, a Mann–Whitney nonparametric test was used to determine statistical significance. When the same vessel was measured multiple times during pressure myography, a repeated measures ANOVA was used. In all other cases, a two‐way ANOVA with post hoc Tukey test was used to determine statistical significance. A ROUT method outlier test (5% aggressive) was used to identify and exclude outliers. The specific test used is indicated in each figure caption. All data are represented as mean ± standard deviation. Statistical significance was determined at *p* < 0.05.

## 3 RESULTS

### 3.1 Glutamine enhanced endothelial cell proliferation in normal and high glucose

**FIGURE 1:**
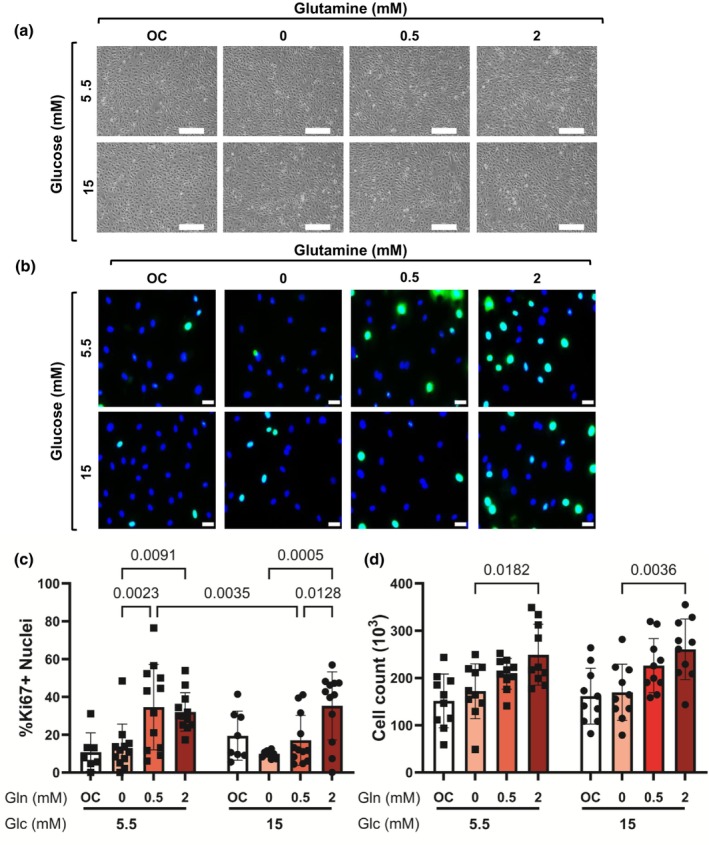
Glutamine increased HCAEC proliferation in normal and high glucose. HCAECs were cultured with 0, 0.5, or 2 mM L‐glutamine in 5.5 and 15 mM glucose for 24 h. L‐glucose at 2 mM (for glutamine in normal glucose) or 11.5 mM (for glutamine and glucose in high glucose) was included as osmotic control (OC). (a) Representative phase contrast images. Scale bar = 500 μm, (b) Representative fluorescent microscopy images (20×). Green = Ki67, blue = nuclei. Scale bar = 100 μm. (c) Quantification of % Ki67+ nuclei. *n* = 12 samples. (d) Cell count after 2 days of incubation in varied glucose and glutamine media. *n* = 10 samples. Data shown as mean ± standard deviation. Statistical significance determined by two‐way ANOVA followed by Tukey's post hoc test.

We first determined the effect of glutamine on endothelial cell proliferation in normal and high glucose culture, since previous studies showed that glutamine increases endothelial cell proliferation in normal glucose culture (Peyton et al., 2018). Phase contrast microscopy images showed similar morphology for HCAECs cultured in 5.5 mM or 15 mM glucose with 0, 0.5, or 2 mM glutamine (Figure 1a). However, glutamine significantly increased endothelial cell proliferation. A two‐way ANOVA showed a significant main effect of glutamine (*p* < 0.0001) and interaction between glutamine and glucose (*p* = 0.0251) on proliferation, with no significant main effect of glucose (*p* = 0.4826). 0.5 and 2 mM glutamine increased proliferating Ki67^+^ cells by ~2.5‐fold compared to 0 mM glutamine in normal glucose culture (*p* = 0.0023 and *p* = 0.0091, respectively; Figure 1b,c). In high glucose culture, Ki67^+^ cells increased by more than 3.5‐fold (*p* = 0.0005) only at 2 mM glutamine relative to 0 mM glutamine (Figure 1b,c). Furthermore, there were twice as many Ki67^+^ cells in normal glucose at 0.5 mM glutamine as compared to in high glucose (Figure 1b,c). To confirm glutamine effects on endothelial cell proliferation, we quantified cell number over 2 days in cells cultured with 0, 0.5, or 2 mM glutamine in normal and high glucose. Two‐way ANOVA only showed a significant main effect of glutamine (*p* < 0.0001) on cell number, with no significant effect of glucose (*p* = 0.4752) or interaction (*p* = 0.9565). We observed approximately 50% more cells in both normal and high glucose in 2 mM as compared to 0 mM glutamine (Figure 1d; *p* = 0.0182 in 5 mM glucose, and *p* = 0.0036 in 15 mM glucose). These data suggest that although endothelial cells maintained their morphology and adhesion in normal and high glucose culture without glutamine, glutamine was required for endothelial cell proliferation in both normal and high glucose.

### 3.2 Glutamine decreased endothelial cell oxidative stress in normal and high glucose

**FIGURE 2:**
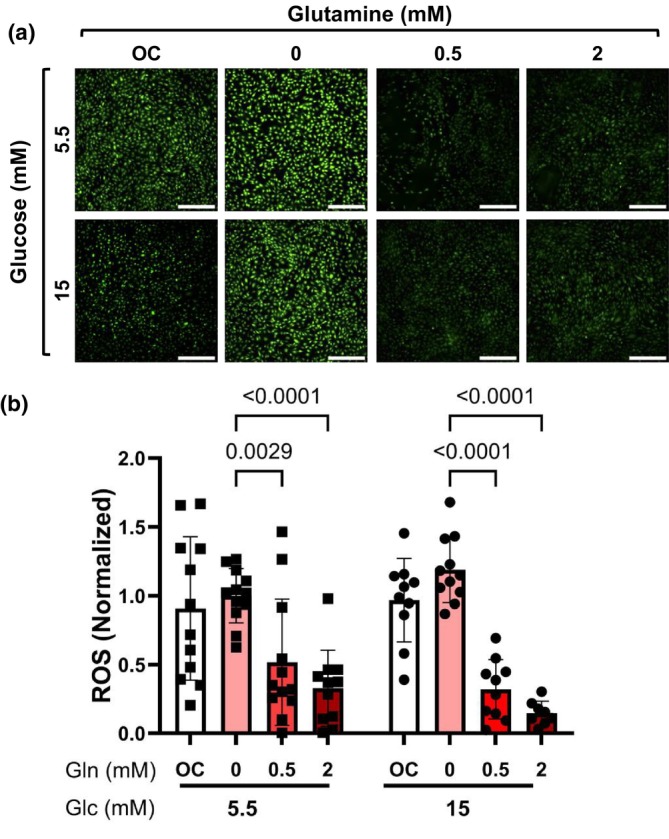
Glutamine decreased oxidative stress. HCAECs were cultured with 0, 0.5, or 2 mM L‐glutamine in 5.5 and 15 mM glucose for 24 h. L‐glucose at 2 mM (for glutamine in normal glucose) or 11.5 mM (for glutamine and glucose in high glucose) was included as osmotic control (OC). (a) Representative confocal microscopy images of HCAEC labeled with 2 μM CM‐H_2_DCFDA (green) with (b) quantification of mean fluorescent intensity. All samples were normalized to 0 mM glutamine in 5.5 mM glucose. *n* = 12 samples, scale bar = 500 μm. Data shown as mean ± standard deviation. Statistical significance determined by two‐way ANOVA followed by Tukey's post hoc test.

Glutamine has previously been shown to decrease endothelial cell oxidative stress in normal glucose (Peyton et al., 2018). To evaluate how glutamine impacts endothelial cell oxidative stress in high glucose, we measured ROS in HCAECs cultured with 0, 0.5, and 2 mM glutamine in normal or high glucose. Two‐way ANOVA indicated a significant main effect of glutamine (*p* < 0.0001) on oxidative stress with no significant effect of glucose (*p* = 0.6570) or interaction (*p* = 0.1548; Figure 2). HCAECs in 0 mM glutamine showed significantly elevated ROS, with more than 2‐fold higher H_2_DCFDA fluorescence compared to HCAECs cultured in 0.5 mM glutamine (*p* < 0.0001) and more than 3‐fold higher compared to HCAECs cultured in 2 mM glutamine (*p* < 0.0001; Figure 2a,b). No significant differences in ROS were observed between HCAEC in normal and high glucose. Thus, these data support the possibility that glutamine reduces endothelial cell oxidative stress in both normal and high glucose conditions.

### 3.3 Glutamine increased endothelial cell survival in oxidative stress in normal and high glucose

**FIGURE 3:**
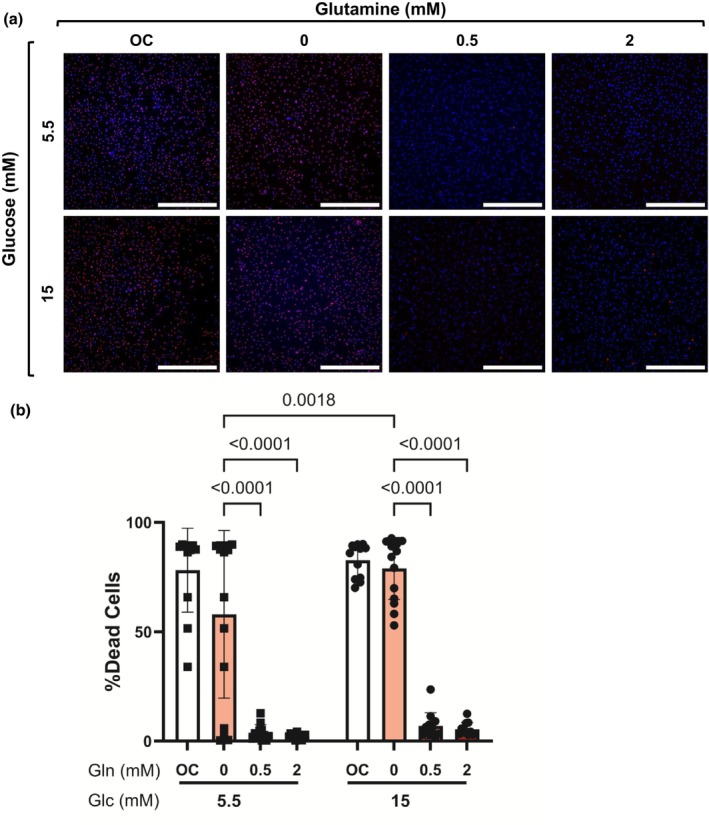
Glutamine increased HCAEC survival in moderate oxidative stress. HCAECs were cultured with 0, 0.5, or 2 mM L‐glutamine in 5.5 and 15 mM glucose for 24 h. L‐glucose at 2 mM (for glutamine in normal glucose) or 11.5 mM (for glutamine and glucose in high glucose) was included as osmotic control (OC). Oxidative stress was induced by adding 50 μM tBHP for 24 h. (a) Representative confocal microscopy images of HCAEC labeled with DAPI (nuclei, blue) and ethidium homodimer‐1 (EthD‐1, dead cells, red). Scale bar = 500 μm. (b) Percentage of dead cells. *n* = 12 samples. Data shown as mean ± standard deviation. Statistical significance determined by two‐way ANOVA followed by Tukey's post hoc test.

We then measured how glutamine impacted endothelial cell survival under oxidative stress. We used 50 μM tBHP for 24 h to induce oxidative stress in HCAECs cultured with 0, 0.5, and 2 mM glutamine in normal and high glucose. Two way ANOVA indicated significant main effects of glutamine (*p* < 0.0001) and glucose (*p* = 0.0283) on cell survival but no significant interaction (*p* = 0.1743; Figure 3). Endothelial cells in 0 mM glutamine showed around 15‐fold higher cell death compared to those treated with 0.5 or 2 mM glutamine (*p* < 0.0001; Figure 3a,b), and this effect was independent of glucose conditions. However, there were statistically significantly more dead cells with 0 mM glutamine in 15 mM glucose culture as compared to 5.5 mM glucose culture (*p* = 0.0018; Figure 3a,b). These data suggest that glutamine is essential for endothelial cell survival during oxidative stress in both normal and high glucose.

### 3.4 Glutamine increased vasodilation without changing eNOS phosphorylation

**FIGURE 4:**
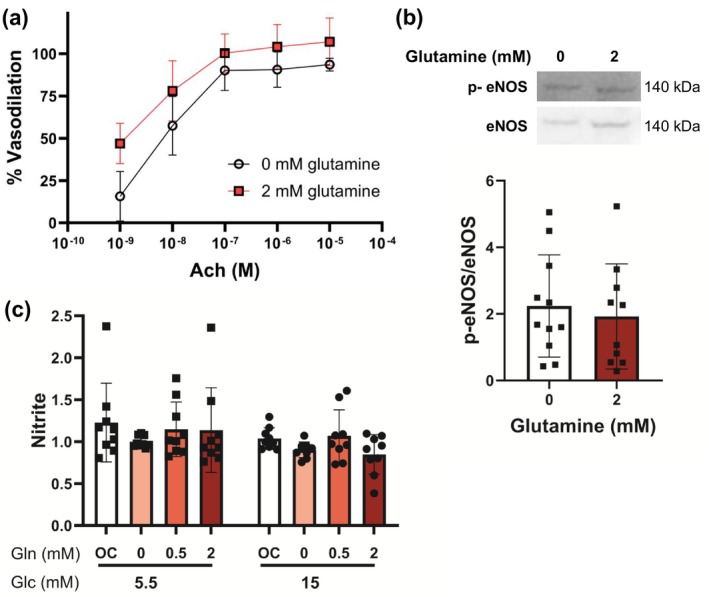
Glutamine increased vasodilation ex vivo but did not impact eNOS phosphorylation or nitric oxide in vitro. (a) Mouse carotid artery vasodilation in response to acetylcholine with an intact endothelium. Isolated mouse carotid arteries were incubated at 4°C overnight in physiological saline containing either 0 or 2 mM glutamine. Vasodilation was measured the following day by pressure myography. *n* = 4–5 arteries per condition. (b) representative Western blot with quantification of eNOS and p‐eNOS in HCAEC cultured in 0 and 2 mM glutamine. *n* = 10–11 samples per condition. (c) HCAECs were treated with 0, 0.5, and 2 mM glutamine in 5.5 and 15 mM glucose. L‐glucose at 2 mM and 11.5 mM was included as positive control for glutamine and glucose respectively. After 24 h cells were treated with 20 μM of yoda1 and then the nitrites in the culture media was measured using a Griess assay. *n* = 9 samples. Data shown as mean ± standard deviation. Statistical significance determined by repeated measures ANOVA (a), Mann–Whitney nonparametric test (b), and two‐way ANOVA followed by Tukey's post hoc test.

ROS can scavenge vasodilatory nitric oxide produced by endothelial cells. We therefore evaluated the impact of glutamine on murine carotid artery vasodilation in response to acetylcholine using pressure myography. Male and female carotid arteries that were incubated with 2 mM glutamine for 24 h showed elevated vasodilation in response to acetylcholine as compared to carotid arteries incubated with 0 mM glutamine by repeated measures ANOVA (*p* = 0.035; Figure 4a and Figure S1a,b). This change was likely nitric oxide dependent, since carotid arteries incubated in both 0 and 2 mM glutamine did not vasodilate when pre‐treated with the eNOS inhibitor L‐NAME (Figure S1c). However, when we measured eNOS phosphorylation in endothelial cells cultured with 0 and 2 mM glutamine in normal glucose, we did not observe any significant changes (*p* = 0.7045; Figure 4b). Two‐way ANOVA indicated a significant effect of glucose on nitrites (*p* = 0.0281; Figure 4c), with nitrites trending lower in 15 mM glucose media at 2 mM glutamine as compared to 5.5 mM glucose media (*p* = 0.0510). However, there was no significant effect of glutamine (*p* = 0.2360) or interaction (*p* = 0.7220) between glucose and glutamine. Thus, glutamine may increase vasodilation without changing eNOS activation or nitric oxide production.

### 3.5 Glutamine increased total endothelial cell glutathione and NADPH in normal and high glucose

**FIGURE 5:**
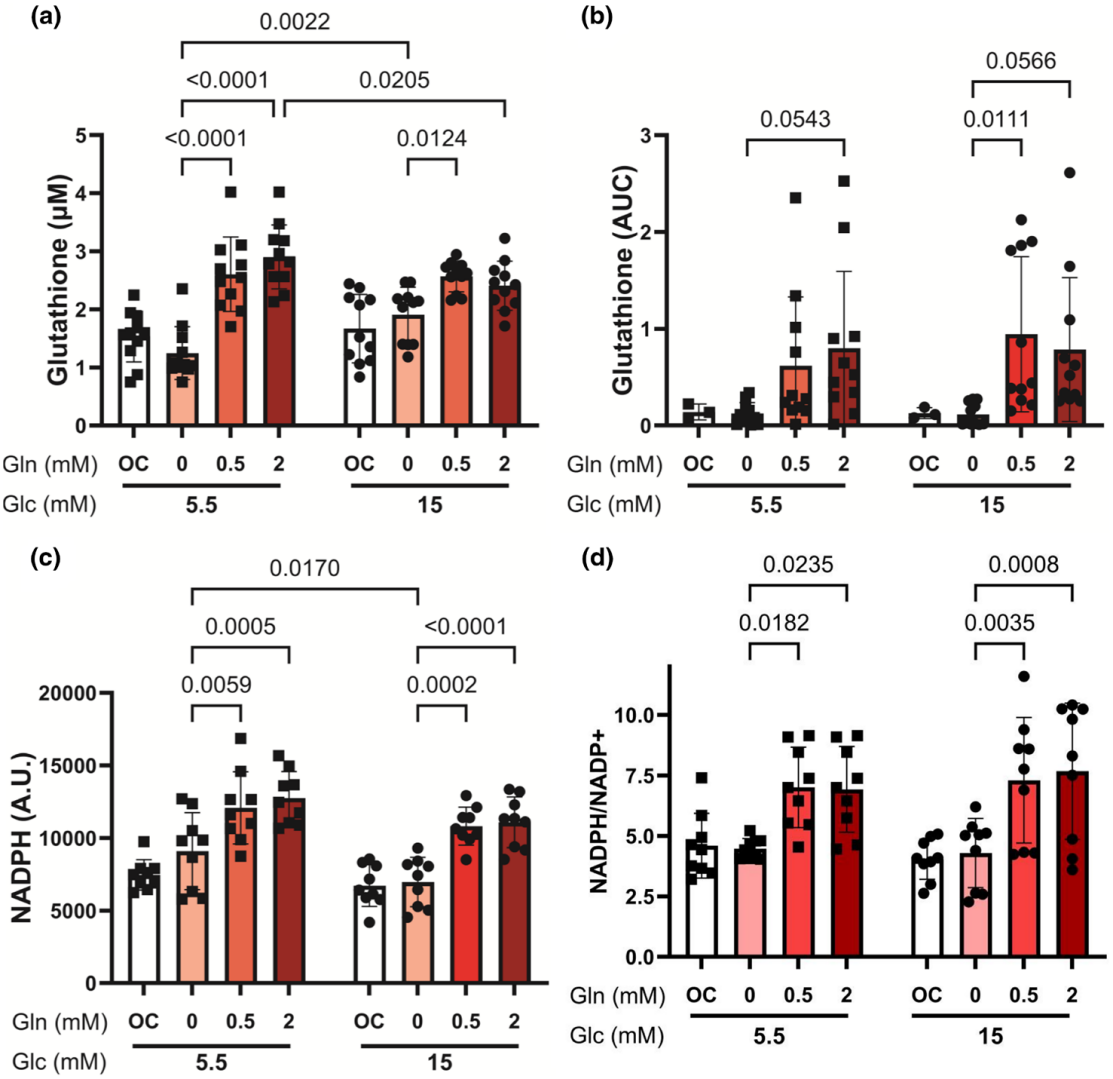
Glutamine increased endothelial cell antioxidants, including glutathione and NADPH. HCAECs were cultured in 5.5 and 15 mM glucose with 0, 0.5, or 2 mM L‐glutamine for 24 h. L‐glucose at 2 mM (for glutamine in normal glucose) or 11.5 mM (for glucose and glutamine in high glucose) was included as osmotic control (OC). (a) Total glutathione concentration measured using GSH‐GSSG Glo assay (*n* = 11 samples). (b) Glutathione area under the curve (AUC) measured by LC‐MS (*n* = 11 samples). (c) NADPH measured using NADPH/NADP+ Glo assay (*n* = 12 samples). (d) NADPH/NADP+ ratio was calculated and normalized to its value at 5.5 mM glucose and no glutamine for each experiment (*n* = 12 samples). Data shown as mean ± standard deviation. Statistical significance determined by two‐way ANOVA followed by Tukey's post hoc test.

The antioxidant glutathione can be derived from glutamine after it is converted to glutamate by GLS1. We therefore measured glutathione in endothelial cells cultured with and without glutamine in normal and high glucose using a GSH/GSSG Glo assay. Two‐way ANOVA indicated a significant effect of glutamine (*p* < 0.0001) and interaction between glucose and glutamine (*p* = 0.0025) on glutathione but no significant effect of glucose (*p* = 0.5301; Figure 5a). In HCAECs cultured under normal glucose, total glutathione increased by more than 2‐fold with both 0.5 mM and 2 mM glutamine compared to 0 mM glutamine (*p* < 0.0001; Figure 5a). In HCAEC cultured in high glucose, total glutathione was 25% higher with 0.5 and 2 mM glutamine as compared to 0 mM glutamine (*p* = 0.0124 for 0.5 mM; Figure 5a). We further found that glutathione was statistically significantly lower in HCAECs cultured in 15 mM glucose and 2 mM glutamine as compared to 5.5 mM glucose (*p* = 0.0205). We validated these findings using LC‐MS. Glutathione trended 5 to 6.5‐fold higher in HCAEC cultured with 0.5 and 2 mM glutamine (*p* = 0.0543) in normal glucose as compared to cells cultured without glutamine (Figure 5b). Similarly, glutathione trended 6 to 8.5‐fold higher in cells cultured with 0.5 and 2 mM glutamine in high glucose compared to cells cultured without glutamine (*p* = 0.0111 and *p* = 0.0566, respectively; Figure 5b).

Since NADPH is a key cofactor for regenerating glutathione from its oxidized form, we measured NADPH in HCAECs cultured in normal and high glucose and varied glutamine. Two‐way ANOVA indicated a significant effect of glutamine (*p* < 0.0001) and glucose (*p* = 0.0015) on NADPH but no interaction (*p* = 0.7069; Figure 5c). NADPH increased 30%–60% in HCAEC cultured with glutamine in both normal and high glucose (*p* = 0.0059 and *p* = 0.0005 for 0.5 and 2 mM glutamine in normal glucose; *p* = 0.0002 and *p* < 0.0001 for 0.5 and 2 mM glutamine in high glucose). NADPH was also statistically significantly higher in HCAEC cultured without glutamine in 5.5 mM glucose as compared to 15 mM glucose (*p* = 0.0170; Figure 5c). We also calculated the NADPH/NADP^+^ ratio, an indicator for cell reducing capacity (Bongard et al., 2009). Two‐way ANOVA indicated a significant effect of glutamine (*p* < 0.0001) but no effects of glucose (*p* = 0.8575) and interaction (*p* = 0.7143; Figure 5d). Glutamine increased the NADPH/NADP+ ratio by more than 50% in cells cultured in both normal and high glucose as compared to 0 mM glutamine (Figure 5d). These results suggest that glutamine increases glutathione and NADPH to enhance ROS scavenging and cellular reducing power in both normal and high glucose.

### 3.6 Glutaminase‐induced glutathione production regulates endothelial cell oxidative stress and survival in normal and high glucose

**FIGURE 6:**
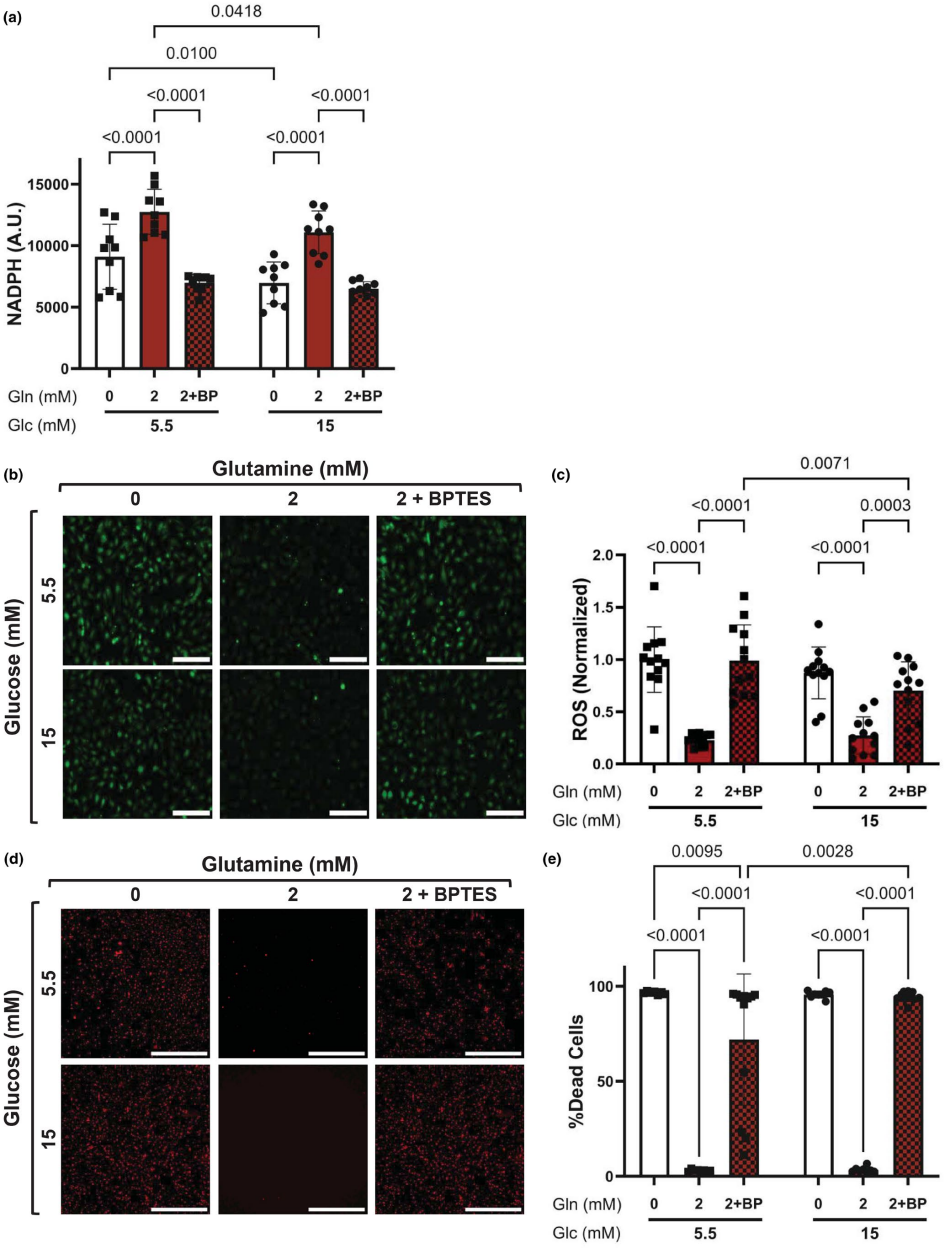
Glutamine‐derived glutathione regulates endothelial reducing capacity, oxidative stress, and survival. HCAECs were cultured 0 or 2 mM glutamine in 5.5 or 15 mM glucose for 24 h. Some cells in 2 mM glutamine were also treated with the glutaminase inhibitor BPTES (BP, 10 μM). (a) NADPH measured using NADPH/NADP+ Glo assay. *n* = 9 samples. (b) Representative confocal microscopy images of HCAECs labeled with ROS indicator CM‐H_2_DCFDA (green). Scale bar = 500 μM. (c) Quantification of ROS mean fluorescence intensity normalized to normal glucose samples cultured with 0 mM glutamine. *n* = 12 samples. (d) Representative confocal microscopy images of HCAECs labeled with DAPI (nuclei, blue) and ethidium homodimer‐1 (EthD‐1, dead cells, red). Scale bar = 500 μm. (e) Percentage of dead cells. *n* = 12 samples. Data shown as mean ± standard deviation. Data were analyzed using ordinary Two‐way ANOVA followed by Tukey's post hoc test.

To determine how glutamine metabolism to glutathione through GLS1 contributes to reduced oxidative stress and enhanced cell survival and vasodilation, we inhibited GLS1 using BPTES (Schoonjans et al., 2020). 10 μM BPTES effectively decreased glutamine uptake, glutamate secretion, and glutathione concentration. BPTES also reduced HCAEC reducing capacity. Two‐way ANOVA indicated a significant effect of BPTES treatment on NAPDH (*p* < 0.0001), a significant effect of glucose (*p* = 0.0031), and no interaction (*p* = 0.3330; Figure 6a). HCAEC NADPH in 2 mM glutamine with BPTES in both normal and high glucose was comparable to NADPH in HCAEC in 0 mM glutamine. BPTES also attenuated glutamine's antioxidant effects. Two‐way ANOVA indicated a significant effect of BPTES treatment (*p* < 0.0001) and glucose (*p* = 0.0427) but no significant interaction (*p* = 0.0833; Figure 6b,c). Glutaminase inhibition in HCAEC cultured in 2 mM glutamine increased ROS to a comparable level as HCAEC cultured in 0 mM glutamine. Finally, the percent of dead cells for HCAEC in 2 mM glutamine and BPTES was comparable to that of HCAEC in 0 mM glutamine in both normal and high glucose (Figure 6d,e). Cell death was higher in BPTES treated cells in 15 mM glucose as compared to 5.5 mM glucose (*p* = 0.0028). Thus, glutamine metabolism to glutamate may regulate NADPH and ROS in endothelial cells and improve their survival under moderate oxidative stress, especially in high glucose.

## 4 DISCUSSION

Glutamine is known to reduce oxidative stress in vitro and in vivo (Das et al., 2007; Nakhostin‐Roohi & Javanamani, 2015); however, its effect on hyperglycemia‐induced oxidative stress in the vasculature, which contributes to cardiovascular complications in diabetes, was not known. We therefore investigated how glutamine impacted endothelial cells cultured in normal and high glucose. Our data suggest that glutamine is required for endothelial cell proliferation and reduces endothelial cell oxidative stress in both normal and high glucose conditions. Glutamine enhanced endothelial cell survival under moderate oxidative stress by increasing intracellular glutathione and NADPH through glutamine deamination to glutamate by GLS1. Glutamine also improved endothelium‐dependent vasodilation ex vivo, likely by reducing oxidative stress. These findings highlight glutamine metabolism, particularly GLS1‐mediated glutathione synthesis, as a critical regulator of endothelial redox balance, which in turn may improve vasodilation by reducing nitric oxide scavenging. Glutamine metabolism may offer a therapeutic strategy to maintain endothelial redox balance under stressful conditions, which could reduce cardiovascular complications associated with diabetes.

Our data expand upon prior studies by supporting that glutamine promotes endothelial cell proliferation in both normal and high glucose. Although endothelial cells are highly glycolytic, most TCA cycle carbons come from glutamine. Both glutamine deprivation and glutaminase inhibition reduced human umbilical vein endothelial cell proliferation in vitro and murine angiogenesis in vivo by preventing the formation of TCA cycle intermediates and nonessential amino acids such as asparagine (Huang et al., 2017; Kim et al., 2017). Peyton et al. (2018) further showed that glutamine‐derived asparagine was essential for cyclin A expression and cell cycle progression in human umbilical vein, aortic, and microvascular endothelial cells in normal glucose. Our findings extend these observations to primary human coronary artery endothelial cells and suggest that glutamine is essential for endothelial cell proliferation even under conditions of excess glucose.

Our data also indicate that glutamine reduced oxidative stress in both normal and high glucose conditions and that this effect was mediated through glutamine deamination by GLS1 into glutamate and ammonia. Others had previously shown that glutamine deprivation and GLS1 inhibition increased endothelial oxidative stress via reduced ammonia, which then decreased expression of antioxidant heme‐oxygenase‐1 (HO‐1) (Liu et al., 2017; Peyton et al., 2018). However, the other product of glutamine deamination, glutamate, is converted to the antioxidant glutathione by glutamate‐cysteine ligase (GCL) and glutathione synthetase (GS) (Zhang & Forman, 2012). Our data demonstrate that glutamine deprivation and glutaminase inhibition also decrease glutathione. Interestingly, both HO‐1 and glutathione are regulated by the transcription factor nuclear factor erythroid 2‐related factor 2 (Nrf2). Nrf2 binds to antioxidant response elements in the HO‐1 promoter to upregulate its transcription, and it additionally increases GCL transcription (Huang et al., 2013; Li et al., 2009).

We also measured decreased NADPH in endothelial cells deprived of glutamine or with inhibited GLS1. NADPH acts as a reducing agent for glutathione reductase, which converts oxidized glutathione back to its reduced form. Glutamine can increase NADPH formation in several ways. After its initial conversion to glutamate, glutamine can be converted to α‐ketoglutarate and eventually to malate in the mitochondrial TCA cycle. This malate is then transported to the cytosol, where malic enzyme 1 (ME1) catalyzes its conversion to pyruvate, a reaction that reduces NADP^+^ to NADPH. Glutaminase inhibition with BPTES decreased NADPH regeneration in control cells but did not impact ME1 knockout cells, linking glutamine metabolism via ME1 to NADPH production (Ying et al., 2021). Alternatively, glutamine‐derived α‐ketoglutarate can be converted to citrate and then isocitrate in the reverse TCA cycle. Isocitrate is then exported to the cytosol, where isocitrate dehydrogenase 1 (IDH1) regenerates α‐ketoglutarate while producing NADPH (Ying et al., 2021). Nrf2 can also increase NADPH‐related gene expression, including ME1 and IDH1 (Lin et al., 2016). Thus, Nrf2 may be a common pathway by which glutamine enhances endothelial antioxidant capacity.

In addition to increasing cell survival, glutamine enhanced acetylcholine‐mediated vasodilation of murine carotid arteries. Glutamine has previously been shown to enhance vasodilation in both animals and humans. In mice with endothelial dysfunction induced by L‐Nω‐methyarginine (L‐NMMA), supplementation with glutamine for 2 months reversed impaired aortic ring vasorelaxation (Addabbo et al., 2013). Similarly, 6 months of β‐hydroxy‐β‐methylbutyrate, glutamine, and arginine supplementation increased flow‐mediated vasodilation by 27% in older adults (Ellis et al., 2016). Epidemiological studies further report that higher plasma glutamine concentration or a higher glutamine:glutamate ratio is associated with reduced CVD incidence and mortality (Chen et al., 2023; Durante, 2019). Our data support these studies and indicate that glutamine supplementation improves vascular function even in a healthy mouse model.

Intriguingly, glutamine enhanced vasodilation but did not impact eNOS phosphorylation (p‐eNOS) or nitric oxide production in cultured endothelial cells. Prior studies showed that glutamine decreased endothelial cell nitric oxide synthesis, in part by increasing glucose flux through the HBP to O‐GlcNAcylate eNOS and prevent its phosphorylation at Ser‐1177 (Aulak et al., 2020; Hecker et al., 1990; Meininger & Guoyao, 1997; Sessa et al., 1990; Wu et al., 2001). Our different results could come from a change in cell type or culture conditions. In our hands, without a change in eNOS or nitric oxide in vitro, our ex vivo increase in vasorelaxation is likely to have resulted from decreased ROS scavenging nitric oxide.

Interestingly, we did not detect an increase in ROS with high glucose culture alone, which has been shown in other studies including those from our laboratory (Kemeny et al., 2013; Serizawa et al., 2011). Our observations may reflect different experimental conditions (e.g., cell type, media, and glucose concentration) or ROS assay sensitivity. We hypothesize that the high glucose conditions we used (15 mM), while physiologically relevant, were not high enough to induce oxidative stress in our cells over short‐term culture, as compared to other studies that used extremely high glucose concentrations (>25 mM). We did observe some detrimental effects of our high glucose culture on the endothelial cells, including decreased cell proliferation, increased cell death, and decreased glutathione and NAPDH at specific glutamine concentrations. Therefore, even though 15 mM glucose did not increase endothelial oxidative stress, it did have negative effects on endothelial cell function and oxidative capacity.

While our study suggests that glutamine regulates ROS in both normo‐ and hyperglycemia and may have cardiovascular protective effects, it is not without limitations. We did not incorporate hemodynamic stimuli on the endothelial cells (e.g., shear stress) or other vascular changes in diabetes such as increased inflammatory cytokines and free fatty acids [Bergman_2007, Ngwa, 2009, Li_2016]. Prior to clinical translation, these data would need to be validated in a more complex diabetic model to confirm the beneficial impact of glutamine.

In summary, our study expands upon previous work by suggesting that GLS1‐dependent glutamate production from glutamine supports endothelial cell glutathione biosynthesis and NADPH generation, which is essential for maintaining endothelial survival and vasodilation in both normo‐ and hyperglycemic conditions. Future research should examine glutamine in the context of other metabolites and disease states to clarify when supplementation may benefit vascular health. Given the endothelial dysfunction observed in diabetes, targeting glutamine metabolism may represent a promising strategy to restore vascular health and prevent diabetic cardiovascular complications.

## AUTHOR CONTRIBUTIONS

MK conceived and designed, performed, and coordinated experiments, analyzed data, interpreted results, wrote the manuscript, and created figures. GS and CMS isolated murine arteries, performed pressure myograph experiments, and analyzed the data. MS performed the live/dead experiment and analyzed the data. XZ performed the isolation of metabolites and LC‐MS experiments for glutathione and analyzed data. LVS performed the Nitrite/Nitrate experiment and analyzed the data. MB developed the LC‐MS method for glutathione detection. AMC oversaw all the experimental procedures, data analysis, and interpretation as well as preparation of the figures and manuscript. All authors contributed to the article and approved the submitted version.

## FUNDING INFORMATION

This work was supported by the National Institutes of Health NIH R01HL165193 and R01HL140239 to A.M.C.

## ETHICS STATEMENT

All animal experiments were approved by the Institutional Animal Care and Use Committee (IACUC) of the University of Maryland (IRBNet ID: 1932985‐6). Human primary endothelial cells were obtained from commercial sources and used in accordance with the suppliers' ethical guidelines. No human subjects were used in this study.

## Supporting information


Figure S1.

## Data Availability

All the supporting data for this study are included in the article and its Figure S1. Additional data are available from the corresponding author upon request.
