# Supplementary material for: Glutamine enhances endothelial cell survival and vasodilation by increasing glutathione to reduce oxidative stress
Source: Physiol Rep. 2026 Jan 21;14(2):e70737. doi: 10.14814/phy2.70737 (PMC12824527; doi:10.14814/phy2.70737)
Supplement: Supplementary file 1 — Figure S1. [file PHY2-14-e70737-s001.docx]

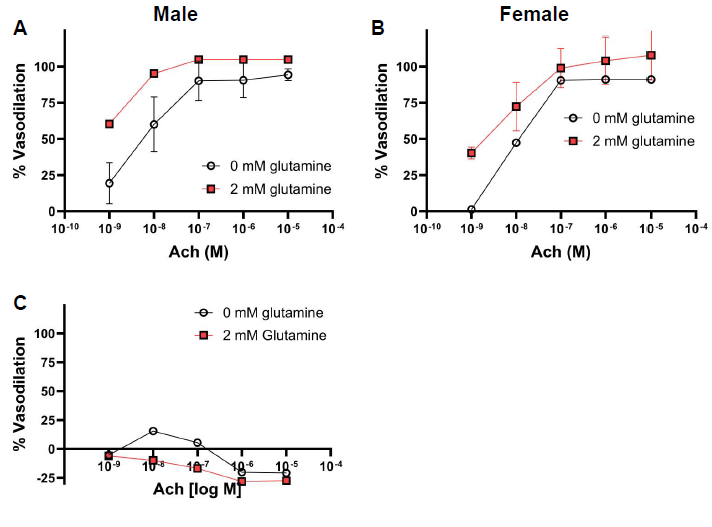


**Figure S1. Glutamine increased vasodilation in male and female arteries and in an NO-dependent manner.** Mouse carotid artery vasodilation in response to acetylcholine with an intact endothelium. Isolated male (A) and female (B) mouse carotid arteries were incubated at 4°C overnight in physiological saline containing either 0 or 2 mM glutamine. For male mice, 0 mM glutamine: n = 4 arteries; 2 mM glutamine: n = 1 artery. For female mice, 0 mM glutamine: n = 1 artery; 2 mM glutamine: n = 4 arteries. Vasodilation was measured the following day by pressure myography. C) Mouse carotid artery vasodilation in response to acetylcholine after eNOS was inhibited using 10^-5^ M L-NAME, as measured by pressure myography. n = 1 artery per condition.
